# Molecular targets for therapy in systemic sclerosis

**DOI:** 10.1186/1755-1536-5-S1-S19

**Published:** 2012-06-06

**Authors:** Naoki Iwamoto, Oliver Distler

**Affiliations:** 1 Department of Rheumatology, Center of Experimental Rheumatology, University Hospital Zurich, Gloriastrasse 25, 8091 Zürich, Switzerland

## Abstract

Despite significant advances have been made in the recent years regarding organ-specific therapies, there is no approved 'disease-modifying' antifibrotic drug for systemic sclerosis (SSc) available to date. Although non-selective immunosuppressive agents are routinely used to treat patients with SSc, large well-controlled studies are lacking for almost all immunosuppressive agents and further evidence is required for long-term beneficial effects of these drugs. Considering these facts about immunosuppressive agents in SSc and also considering the high mortality of SSc, other therapeutic strategies are urgently needed. Recently an important role of the 5-hydroxytryptamine (5-HT: serotonin) pathway in fibrosis was reported. In this review, we discuss the role of 5-HT in fibrosis and therapeutic potential of this molecule. Besides 5-HT, there are a number of promising targets that have been extensively characterized in recent years. For many of these molecular targets, modifiers are readily available for clinical studies, and often these modifiers are used already in clinical use for other diseases. Results from these studies will show, in how far the promising preclinical results for novel antifibrotic strategies can be translated to clinical practice.

## Introduction

Systemic sclerosis (SSc) is a multisystem connective tissue disease that is characterized by fibrosis of the skin and internal organs and also by widespread vasculopathy. Clinical manifestations include thickening of the skin, Raynaud's phenomenon, pulmonary arterial hypertension, pulmonary fibrosis and involvement of other visceral organs [1]. Histological hallmarks in the skin of early stage of SSc are perivascular inflammatory infiltrates and microvascular changes such as capillary dilatation with subsequent rarefaction. In later stage, this leads to tissue fibrosis with an excessive accumulation of extracellular matrix [2,3]. Tissue fibrosis disrupts the physiological tissue architecture and causes dysfunction of the affected organs. Organ dysfunction can lead to organ failure and contributes profoundly to the increased mortality of SSc. Despite intensive research, lack of evidence for the treatment of SSc patients complicates the appropriate management of SSc patients.

Although its etiology still remains unknown, knowledge about the pathogenesis of SSc is rapidly increasing. Significant progress has been achieved in the identification of possible molecular targets for therapy in SSc. In this article, we will summarize current therapeutic approaches using immunosuppressive agents in SSc, discuss limitations of such approaches and focus on novel molecular targets for therapy to treat fibrotic manifestations by using the 5-hydroxytryptamine (5-HT: serotonin) pathway as an example.

## Current therapy for SSc

Significant advances have been made in the recent years regarding symptomatic organ-specific therapies [4]. However, there is no approved 'disease-modifying' drug for SSc that modifies the fibrotic manifestations of the disease.

Non-selective immunosuppressive agents are routinely used to treat patients with SSc. A recent report from the German SSc registry showed that 41% patients received corticosteroids and 36% received immunosuppressive agents [5]. Despite their frequent use, there is only limited data on their efficacy in SSc and only few controlled clinical studies have been performed for nonselective immunosuppressive agents. The low prevalence of SSc, various disease subsets and a highly variable course of the disease are making it difficult to perform well-designed clinical studies with a significant number of patients [6]. Table 1 summarizes the main randomized controlled trials (RCTs) that have been performed to date.

**Table 1:** Randomized controlled trials evaluating immunosuppressive/immunomodulatory drugs in patients with SSc.

Reference	Treatment	Number and main inclusion criteria of SSc patients	Study duration	Clinical effect
Furst DE et al. Arthritis Rheum 1989;32:584	Chlorambucil p.o. 0.05-0.1 mg/kg/day versus placebo	65 SSc	3 years	NS effect

O'Dell JR et al. J Rheumatol 1989;32:584	Total lymphoid irradiation versus untreated control	6 SSc with internal organ involvement	Follow-up of 1-4 years	NS effect

Casaes JA et al. Ann Rheum Dis 1990;49:926	5-fluorouracil i.v. 4 × 12 mg/kg daily, followed by 4 × 6 mg/kg every two days and maintenance therapy with 12.5 mg/kg weekly versus placebo	70 SSc (diffuse or limited with visceral involvement)	6 months	Significant improvement in skin score, Raynaud's score and patient's general assesment scores

Sharada B et al. Rheumatol Int 1994;14:91	Dexamethasone i.v. 100 mg/month versus placebo	35 diffuse SSc	6 months	Significant improvement in skin score

Van den Hoogen FH et al. Br J Rheumatol 1996;35:364	Methotrexate i.m. 15 mg/week versus placebo	29 SSc with <3 years of skin involvement or with disease progression	24 weeks	Trend towards improvement in skin score (p = 0.06 in comparison with placebo)

Clements PJ et al. Arthritis Rheum 2001;44:1351	D-penicillamine p.o. high (750-1000 mg/d) versus low (125 mg every second day) dose	134 early diffuse SSc	2 years	NS effect

Pope JE et al. Arthritis Rheum 2001;44:1351	Methotrexate p.o. 15 mg/week versus placebo	71 early diffuse SSc	1 year	Improvement in skin scores, borderline significance

Tashkin DP et al. N Eng J Med 2006;354:2655	Cyclophosphamide p.o. 1-2 mg/kg/d versus placebo	154 SSc with early (<7 years duration) SSc, symptomatic SLD and alveolitis	1 year	Significant improvement in lung volumes, dyspnoea and some measures of health related quality of life. Significant improvement in skin score in diffuse SSc as a secondary outcome measure

Hoyles Rk et al. Arthritis Rheum 2006;54:3962	Cyclophosphamide 6 × i.v. 600 mg/m^2^/month plus oral predonisone 20 mg every second day followed by azathioprine (p.o. 2.5 mg/kg/day) versus placebo	45 SSc with SLD	1 year	Trend towards improvement of FVC (p = 0.08), but low power

Nadashkevich O et al. Clin Rheumatol 2006;25:205	Cyclophosphamide p.o. 2 mg/kg/d for 12 months, then 1 mg/kg/d for another 6 months versus asathioprine 2.5 mg/kg/d for 12 months and then 2 mg/kg/d for 18 months	60 early diffuse SSc (<12 months duration)	18 months	Significant improvement in skin score, lung function tests and frequency of attacks of Raynaud's phenomenon

i.v. = intravenous, i.m. = intramuscular, p.o. = peroral, NS = not significant, SLD = scleroderma interstitial lung diseases, SSc = systemic sclerosis. This list is not complete and is only a selection of potential molecules for targeted therapies. Modified from Distler O: Screloderma-modern aspects of pathogenesis, diagnosis and therapy. UNI-MED 2009.

Although there is clinical evidence that corticosteroid treatment is effective for some clinical manifestations that can occur in association with SSc such as inflammatory arthritis and myositis, use of higher dose corticosteroids is not recommended in SSc except for certain treatment-resistant and severe cases. The reason for this recommendation is that several retrospective case-control studies revealed that corticosteroid doses with a prednisone equivalent of more than 15 mg/day increased significantly the risk of developing scleroderma renal crisis, which is a severe and life threatening disease manifestation of SSc [7,8].

Large well-controlled studies are lacking for almost all immunosuppressive agents that are used in SSc. However, few randomized RCTs for immunosuppressive therapies are available. A beneficial effect on skin fibrosis was shown for methotrexate, 5-fluorouracyl, oral cyclophosphamide and intravenous dexamethasone. This effect was mainly seen in patients with diffuse SSc and the effects were small. It is questionable whether these effects are clinically meaningful. Beneficial effects on internal organ involvement were only seen in patients with scleroderma lung disease treated with oral cyclophosphamide showing improved lung volumes and dyspnoea scores compared to placebo. Again, the observed effects were small, and all effects seen with cyclophosphamide treatment, except for sustained improvement in patient-reported dyspnoea, waned within 12-18 months after stopping therapy [9]. Several other immunosuppressive medications including mycophenolate mofetil, anti-thymocyte globulin, azathioprine, cyclosporine, intravenous immunogloblins or combination of cyclophosphamide with high dose corticosteroids have shown some efficacy in small open-label, uncontrolled studies or retrospective analyses, but well-performed RCTs are not available.

In summary, small observational trials, case reports and a limited number of RCTs suggested some efficacy of immunosuppressive agents. Thus, an immunosuppressive therapeutic approach may be considered in early inflammatory stages of diffuse SSc. However, further evidence is required for long-term beneficial effects of these drugs. In addition, the observed effects in clinical trials were often small, which also reflects the experience in clinical practice. The potential toxicity of immunosuppressive treatments also needs to be included in a risk/benefit analysis and may limit long-term usage of these treatments in SSc. Considering the high mortality of SSc secondary to fibrotic manifestations, other therapeutic approach are urgently needed for SSc.

## Novel therapeutic target for fibrosis in SSc

Indeed, significant inflammatory infiltrates in the skin are often limited to very early disease stages, and even in these early disease stages meaningful infiltrates can only be found in perivascular areas. There is also increasing evidence that the molecular expression pattern in SSc skin is extremely heterogeneous, and that only smaller subsets of patients express increased levels of inflammatory molecules [10]. Thus, unselective immunosuppressives might be useful for smaller subsets of SSc patients, while the large majority of patients might need different therapeutic approaches. In this regard, mechanisms leading to the increased synthesis of extracellular matrix proteins in fibroblasts are of particular interest for targeted therapies.

Remarkable breakthrough findings have been obtained regarding the identification of key molecules, key cellular mechanisms, and key intracellular signaling cascades, which mediate the perpetuation of fibrosis. These findings have true translational implications, because the modifiers of these key mediators and key mechanisms are often in clinical use in other diseases such as cancer. Among these key molecules, recent reports showed an important role of the 5-hydroxytryptamine (5-HT: serotonin) pathway in fibrosis [11]. Here, we focus on the serotonin pathway as a novel target in the treatment of SSc and summarize these recent results as an example for a preclinical characterization of molecular targets in SSc.

Microvascular damage is one of the earliest features in the pathogenesis of SSc leading to a progressive loss of capillaries [12]. Microvascular injury precedes clinically detectable tissue fibrosis in SSc. However, molecular links between microvascular damage and the induction of tissue fibrosis have not been established. One possible link could be 5-HT, because the microvascular damage with exposure of subendothelial connective tissue activates platelets and activated platelets release large amounts of 5-HT. Based on these considerations and the fact that 5-HT is elevated in the blood of SSc patients [13-15], we hypothesized that 5-HT signaling might be involved in the process of fibrosis in SSc [11].

First, we investigated, whether 5-HT is able to induce the release of extracellular matrix proteins in SSc. Primary dermal fibroblasts from SSc patients and healthy subjects, which were stimulated with 5-HT, increased the mRNA of different extracellular matrix proteins such as collagen type I alpha 1 (COL1A1), collagen type I alpha 2 (COL1A2) and fibronectin-1 in dose-dependent manner. Similarly, the release of collagen protein was also increased by 5-HT stimulation. Doses used for the stimulations were in the range of those detected in biological fluids.

There are seven 5-HT receptors, 5-HT1 to 5-HT7, and the cellular effects of 5-HT are mediated by these receptors. The identification of those receptors, which play a crucial role in fibrosis, was necessary, because receptor antagonists often show more efficacy and safety than ligand antagonist. Thus, we next investigated, which receptor specifically mediates the profibrotic effects of 5-HT. We observed that three different 5-HT receptors were expressed by dermal fibroblasts: 5-HT_1B_, 5-HT_2A_, and 5-HT_2B_. The mRNA levels of 5-HT_2B _were up regulated in SSc fibroblasts as compared with healthy controls (143 ± 17%), while 5-HT_1B _and 5-HT_2A _were not different. Inhibition of 5-HT1 by selective chemical inhibitors showed no reduction of the stimulatory effects of 5-HT on extracellular matrix production in SSc fibroblasts. In contrast, inhibition of 5-HT2 decreased mRNA levels of COL 1A1, COL 1A2 and fibronectin-1 and also decreased the release of collagen protein. Additional inhibition experiments using chemical inhibitors as well as siRNA approaches revealed that this effect was specific for 5-HT_2B_, but not for 5-HT_2A_. These results suggested that 5-HT_2B _plays crucial role for the synthesis of extracellular matrix proteins in dermal fibroblasts.

Immunohistochemistry in fibrotic skin biopsies of SSc patients and normal skin of healthy individuals backed up these results. Expression of 5-HT_2B _was strongly increased in fibrotic tissue as compared with unaffected tissue from healthy controls. Double staining with the fibroblast-specific marker prolyl-4-hydroxylase-beta confirmed that 5-HT_2B _was mostly expressed by dermal fibroblasts, and the large majority of fibroblasts stained positive for 5-HT_2B _in fibrotic tissue, but not in controls.

The profibrotic effects of 5-HT/5-HT_2B _could be mediated via direct increase of collagen mRNA transcription or indirectly via induction of a second mediator. We suspected indirect mechanisms, because the effects of 5-HT in the COL1A2 reporter assay were delayed compared to the effects of transforming growth factor-β (TGF-β), which is a well known key player in the pathogenesis of fibrosis [16]. Thus, we investigated, whether TGF-β itself might be the second mediator of 5-HT signalling. Interestingly, 5-HT increased dose-dependently mRNA levels of TGF-β1 in SSc fibroblasts. Furthermore, 5-HT induced in a time-dependent manner the nuclear levels of phospho-Smad3, the typical intracellular mediator of TGF-β signalling. To evaluate whether TGF-β is necessary for the profibrotic effect of 5-HT, we cultured SSc fibroblasts with neutralizing antibodies against TGF-β1. Inhibition of TGF-β1 completely abrogated the profibrotic effects of 5-HT on mRNA expression of COL1A1, COL 1A2 and fibronectin-1.

Next, we aimed to demonstrate that these in vitro results reflect in vivo situations by using the mouse model of bleomycin-induced dermal fibrosis. Injection of bleomycin potently stimulated the expression of 5-HT_2B _and induced dermal fibrosis. The 5-HT2 inhibitors terguride and cyproheptadine as well as the selective 5-HT_2B _inhibitor SB 204741 [17-20] efficiently prevented bleomycine-induced dermal thickening. Collagen content and myofibroblast counts were also reduced dose-dependently by inhibition of 5-HT_2B_. The antifibrotic effects of 5-HT2 inhibition were further tested in a therapeutic approach using a modification of the bleomycin model. Dermal thickening decreased by 78 ± 4% in mice treated with terguride for the last 3 weeks compared with placebo treated mice. Finally, we used 5-HT_2B _-deficient mice to confirm the role of 5-HT_2B _in experimental fibrosis. 5-HT_2B_^-/- ^mice were almost completely protected from bleomycin-induced dermal fibrosis, although no spontaneous histological changes or differences in dermal thickness were observed in untreated 5-HT_2B_^-/- ^mice.

There is no single animal model that completely covers the different aspects in the pathogenesis of SSc [21]. For example, in the bleomycin model, fibrosis is triggered by intense inflammatory infiltrates in the skin. As outlined above, inflammatory infiltrates are present in early inflammatory stages of human SSc, but are not a feature of later disease stages. Thus, for the identification of potential molecular targets for therapy, testing in at least one additional model is recommended [22]. We therefore assessed the role of 5-HT_2B _in a less inflammation-dependent mouse model of fibrosis, the tight skin 1 (Tsk-1) model. As in human SSc and experimental bleomycin-induced fibrosis, 5-HT_2B _was overexpressed in skin sections of Tsk-1 mice. Hypodermal thickening, collagen content, and differentiation of resting fibroblasts into myofibroblasts were significantly reduced in Tsk-1 mice upon treatment with the 5-HT_2B _inhibitor SB 204741. Similarly, Tsk-1 mice crossed with 5-HT_2B_^-/- ^mice showed reduced hypodermal thickening, reduced collagen content, and decreased numbers of myofibroblasts compared with TSk-1 mice crossed with 5-HT_2B _^+/+ ^mice.

To confirm the link between platelet activation and increased 5-HT/5-HT_2B _signalling, we examined whether inhibition of platelet activation reduces tissue levels of 5-HT and prevents experimental fibrosis. Indeed, treatment with the P2Y_12 _receptor inhibitor clopidogrel reduced the content of 5-HT in fibrotic skin of bleomycin-challenged mice by 58 ± 21%. In parallel, clopidogrel decreased dermal thickening by 61 ± 13% as compared with control mice. This was also seen in Tsk-1 mice experiments, in which treatment with clopidogrel reduced hypodermal thickening by 51 ± 16%.

Tryptophan hydroxylase (TPH) 1 is the key enzyme for the synthesis of 5-HT in platelets. Experiments with TPH 1 deficient mice further underlined the important role of platelet-derived 5-HT in experimental fibrosis. Blood levels of 5-HT in TPH1^-/- ^mice were reduced to 5% compared with wild-type animals [23]. Dermal thickening in bleomycin-challenged TPH1^-/- ^mice were decreased by 61 ± 6% as compared with bleomycin-challenged TPH1^+/+ ^mice. Similarly, collagen content and myofibroblast counts were significantly reduced.

## Conclusion

These data provide circumstantial evidence for 5-HT/5-HT_2B _signalling as a potential therapeutic target in SSc. They also provide a template for an extended pre-clinical characterization of other potential therapeutic targets in fibrotic diseases such as SSc. Indeed, there are a number of promising targets that have been extensively characterized in recent years, and 5-HT/5-HT_2B _is just one example among them. Table 2 gives an overview of potential targets for therapy in SSc. Most interestingly, for many of these molecular targets, modifiers are readily available for clinical studies, and often these modifiers are used already in clinical practice for other diseases. The 5-HT inhibitor terguride is approved in Japan for the treatment of hyperprolactinemia and is currently tested in a proof of concept trial in patients with SSc. This study has finished recruitment and is currently awaiting analysis. This and similar studies will show, in how far the extended preclinical characterization outlined above is able to predict clinical responses in human fibrotic diseases such as SSc.

**Table 2:** Possible molecular targets for therapy in SSc

Target molecules	Available drug name	References
Tyrosine kinases	Imatinib, Nilotinib, Dasatinib	Iwamoto N et al. Curr Rheumatol Rep. 2011;13:21

SRC kinases	SU6656, Dasatinib	Akhmetshina A et al. FASEB J. 2008;22:2214

TGF-b inhibitors	Several drugs	Asano Y. J Dermatol 2010;37:54

Histondeacetylase inhibitor	Trichostatin, SAHA	Huber LC et al. Arthritis Rheum 2007;56:2755

DNA Methyltransferase inhibitors	5-AZA	Wang Y et al. Arthritis Rheum 2006;54:2271

Rho associated kinase	Fasudil	Akhmetshina A et al. Arthritis Rheum 2008;58:2553

Cannabinoid receptor 2 (CB2) agonists	Several drugs	Akhmetshina A et al. Arthritis Rheum. 2009;60:1129

PPAR γ agonists	None	Wei J et al. PloS One 2010;5:e13778

Adenosine A2A receptor blockers	None	Chan ES et al. Arthritis Rheum. 2006;54:2632

IL-13 inhibitors	None	Lafyatis R et al. Endocr Metab Immune Disord Targets 2006;6:395

Serotonin-Receptor-2B inhibitors	Terguride	Dees C et al. J Exp Med. 2011; 208:961

CTGF inhibitor	CTGF antibodies	Wang Q et al. Fibrogenesis Tissue Repair 2011;4:4

IL-6 receptor antagonist	Tocilizmab	Shima Y et al. Rheumatology(oxford) 2010;49:2408

MicroRNA-29a	None	Maurer B et al. Arthritis Rheum 2010;62:1733

Fos-Related Antigen-2 (AP-1 family)	None	Reich N et al. Arthritis Rheum 2010;62:280 Maurer B et al. Circulation. 2009;120:2367

## List of abbreviations used

SSc: Systemic sclerosis; RCTs: randomized controlled trials; 5-HT: 5-hydroxytryptamine; COL1A1: collagen type I alpha 1; COL1A2: collagen type I alpha 1; TGF-β:transforming; Tsk-1: tight skin 1; TPH: tryptophan hydroxylase.

## Competing interests

Dr. O. Distler has received consulting fees and/or research grants from Pfizer, Actelion, Encysive, FibroGen, Ergonex, NicOX, Bristol-Myers Squibb, Sanofi-Aventis, United BioSource, Medac, Biovitrium and Active Biotech with regard to potential scleroderma treatments.
